# Imaging trace element distributions in single organelles and subcellular features

**DOI:** 10.1038/srep21437

**Published:** 2016-02-25

**Authors:** Yoav Kashiv, Jotham R. Austin, Barry Lai, Volker Rose, Stefan Vogt, Malek El-Muayed

**Affiliations:** 1 Department of Physics, University of Notre Dame, Notre Dame, IN 46556-5670, USA; 2 Advanced Electron Microscopy Facility, Department of Molecular Genetics & Cell Biology, The University of Chicago, Chicago, IL 60637, USA; 3 X-Ray Science Division, Argonne National Laboratory, Argonne, IL 60439, USA; 4 Center for Nanoscale Materials, Argonne National Laboratory, Argonne, IL 60439, USA; 5 Division of Endocrinology, Metabolism and Molecular Medicine, Northwestern University Feinberg School of Medicine, Chicago, IL 60611, USA

## Abstract

The distributions of chemical elements within cells are of prime importance in a wide range of basic and applied biochemical research. An example is the role of the subcellular Zn distribution in Zn homeostasis in insulin producing pancreatic beta cells and the development of type 2 diabetes mellitus. We combined transmission electron microscopy with micro- and nano-synchrotron X-ray fluorescence to image unequivocally for the first time, to the best of our knowledge, the natural elemental distributions, including those of trace elements, in single organelles and other subcellular features. Detected elements include Cl, K, Ca, Co, Ni, Cu, Zn and Cd (which some cells were supplemented with). Cell samples were prepared by a technique that minimally affects the natural elemental concentrations and distributions, and without using fluorescent indicators. It could likely be applied to all cell types and provide new biochemical insights at the single organelle level not available from organelle population level studies.

The chemical compositions of organelles within cells, and the relationship of the chemical compositions to the organelle morphologies and functions, are key for understanding biochemical processes in healthy and diseased cells. Specifically, the study of the biological roles, buffering, trafficking and compartmentalization of elements, especially Cu and Zn, in eukaryotic cells, under normal and pathologic conditions, is an active area of research at the forefront of biochemistry^1^,^2^,^3^,^4^,^5^,^6^,^7^. However, progress in the field has been hampered by limitations of the techniques commonly used for chemical analysis. These techniques can be divided to two groups, macro-analytical techniques, which analyze aggregates of organelles, and micro-analytical techniques, which analyze single organelles.

Macro-analytical techniques^8^,^9^ involve isolating a large number of organelles of a certain type from cells by lysis and fractionation. The chemical composition of the organelle aggregate is then usually determined by inductively coupled plasma - mass spectrometry (ICP-MS). This analysis represents the average composition of a population of organelles and cannot reveal compositional differences between single organelles. In addition, chemical elements not covalently bonded to atoms in cell structures are especially susceptible to lysis and fractionation and their natural concentrations could be altered significantly in the process.

Most relevant micro-analytical techniques lack the required spatial resolution and/or elemental detection sensitivity to measure the chemical compositions of single organelles, including trace elements, with the exception of the nucleus^10^,^11^. The most widely used technique in this category is fluorescence optical microscopy of cells loaded with fluorescent indicators (that fluoresce when binding to their target elements). In addition to the relatively low spatial resolution of optical microscopy, the technique is limited as well by two properties of the fluorescent indicators: They are not entirely specific to their target elements, and they bind primarily to the free or loosely bound fraction of the target elements in the cells. The latter means that by binding to their target elements, the fluorescent indicators potentially alter the natural elemental distributions.

An example is the fluorescent indicators used for detecting the biochemically essential element Zn. They have moderate Zn binding affinities, which are significantly lower than those of typical Zn-binding enzymes, such as carbonic anhydrase^12^. Thus, the fluorescent indicators detect chelatable Zn that is loosely bound to intracellular Zn-binding proteins that have lower affinities for Zn. However, most cellular Zn is reported to be bound to metallothioneins and other proteins which have much higher affinities for Zn^13^ and therefore cannot be detected by Zn fluorescent indicators. While fluorescent indicators with higher Zn affinity are theoretically feasible, they are likely to scavenge Zn from Zn-binding and transporting proteins, thereby altering the natural distribution of Zn. Therefore, commonly used Zn fluorescent indicators are unable to detect cytosolic Zn, unless labile Zn concentrations exceed the buffering capacity of the abundant buffering proteins^4^,^14^,^15^. The situation is the same for many other biochemically essential elements in cells, e.g., Fe and Cu, which are tightly bound to buffering proteins as well. This property limits the utility of fluorescent indicators for many applications, especially as tracers of elemental distributions in single organelles.

Additional micro-analytical techniques include conventional and scanning transmission electron microscopy (TEM and STEM), and nano-secondary ionization mass spectrometry (NanoSIMS). The former techniques have superb spatial resolution, at the sub-nanometre (nm) level, but their elemental detection limits are not sensitive enough to detect most trace elements. The latter technique has high spatial resolution, down to 50 nm, and high sensitivity, which enables the detection of some trace elements (e.g., P and S). At the subcellular level, it is primarily used to study metabolism in isotopically labeled cells^16^,^17^, hence it could be a complementary technique to the method presented here. Two limitations of NanoSIMS that need to be considered are potential isobaric interferences (i.e., interferences by atoms and/or molecules of the same nominal mass as the isotope of interest) and the fact that it is destructive.

Synchrotron X-ray fluorescence (SXRF) chemical imaging, combined with appropriate sample preparation methods and high spatial resolution structural imaging techniques, is a promising micro-analytical technique for analyzing elemental distributions in single organelles. It measures the chemical compositions of samples without the need for using fluorescent indicators or isotopically labeling the cells, hence it avoids the limitations of biased fluorescent indicator binding and altering the natural elemental distributions. However, like with other analytical techniques, determining elemental concentrations with SXRF may be limited by background and/or elemental interferences. The mostly flat background is due to scattered primary X-ray photons (the tail of the Compton peak). Elemental interference is due to X-ray fluorescence peaks of other elements that partially or fully overlap peaks of the elements of interest. In order to improve elemental detection limits, one takes steps to decrease both sources of interference. Minimizing the scattering background is done by placing the detector in the plane of the synchrotron storage ring at 90° to the primary X-ray beam (scattering is minimized when the angle between the beam electric vector, which is the direction of the beam polarization vector, and the scattering vector is 0°, as in this configuration) and selecting the thinnest possible sample substrate, usually a few 100 nm thick (a thicker substrate would be a major source of scattered primary X-ray photons in thin samples). Minimizing elemental interference is done by using sample preparation procedures that reduce the concentrations of potentially interfering elements in the sample.

Earlier studies with the SXRF microprobe, with spatial resolutions down to ~150 nm, of chemically treated and untreated, chemically fixed, whole cells (a few micrometre (μm) thick) were able to image elemental distributions in some subcellular features^18^,^19^,^20^,^21^. Subcellular structures were imaged as well in thin (300 nm) sections of chemically treated and untreated cells that were prepared by high-pressure freezing^22^.

The introduction of SXRF nanoprobes in recent years^23^, with spatial resolutions in the range of tens of nm, coupled with improved elemental detection sensitivity, brought researchers closer to imaging trace element distributions in single organelles. When nano-SXRF was combined with the relatively low spatial resolution of optical microscopy for structural imaging, the chemical compositions of organelles were imaged in thick (whole cell) sections of chemically treated cells prepared by freeze-drying^24^ or by chemical fixation^25^ (the primary X-ray beam is likely to transverse a number of organelles in thick samples). In a novel study that combined nano-SXRF with X-ray ptychography (for structural imaging), the chemical compositions of organelles were imaged in whole, frozen-hydrated, green algae cells at cryogenic temperatures^26^. In addition, the chemical compositions of isolated single, freeze-dried, melanosomes (organelles where melanin is synthesized, stored and transported) were imaged as well^27^.

We present here, to the best of our knowledge, the first unequivocal imaging of natural trace element distributions in single organelles in cells. This was achieved by combining the highest available spatial resolution nano-SXRF with TEM and improved sample preparation protocol, of minimally chemically treated cells, that minimized the potential for altering the natural elemental distributions. The method opens up new possibilities in subcellular biochemistry for studying variabilities among single organelles and other subcellular compartments, which are usually analyzed at the organelle population level.

## Results

### Analytical techniques

In order to map elemental distributions in single organelles and other subcellular features, we combined the high spatial resolution of TEM, for cellular ultrastructure imaging, with the high spatial resolution and high sensitivity of micro- and nano-SXRF, for chemical imaging. The hard X-rays commonly used in SXRF can detect Al and heavier elements with a detection limit of ~1 part per million (ppm), depending on experimental conditions. In addition, under normal operating conditions, these two techniques are non-destructive, which allows repeated analyses of the same samples.

### Cells and preparation

Cells of the insulin producing mouse pancreatic beta cell line MIN6^28^ were grown for 72 hours in a cell culture medium. For a subset of the cells, the medium was supplemented with 1 μmole/l CdCl_2_ in order to introduce the biochemically non-essential element Cd and examine its intracellular distribution. The cells were then prepared using high pressure freezing, followed by freeze substitution. This technique is preferable to the commonly used chemical fixation in terms of superior preservation of both the cell’s original ultrastructure^29^,^30^ and chemical composition^30^, especially with regard to elements bound to soluble compounds^31^. In order to minimize the possibility of altering the natural elemental distributions in the cells during preparation, especially with respect to biochemically important trace elements, we took a number of additional steps: (1) Cells were only fixed with glutaraldehyde. The commonly used fixer osmium tetroxide (OsO_4_) was specifically avoided to prevent Os Lα_1,2_ lines, with energies 8.912, 8.841 keV, respectively, interfering with the Kα_1,2_ lines of Zn, 8.639, 8.616 keV, respectively^31^. (2) Cells were stained only with tannic acid, which provided contrast for ultrastructure imaging, but does not contain any heavy elements, with potentially interfering elemental X-ray lines, as major components. The commonly used staining materials lead citrate and uranyl acetate were avoided due to interferences of the Pb and U Mα_1_ lines, 2.346 and 3.171 keV, respectively, with the Kα_1,2_ lines of biochemically important S, 2.308 and 2.307 keV, and the Lα_1,2_ lines of Cd, 3.134 and 3.127 keV. (3) Cells were embedded in HM20 Lowicryl resin, which can be used at cryogenic temperatures, and better preserves ultrastructure and intrinsic chemical composition compared with most other resins^30^. (4) To prevent diffusion of atoms out of/into the cells, ethylene glycol was used in ultramicrotoming the cells instead of water^31^. Trace element concentrations in the chemicals used to prepare the samples are given in Supplementary Table S1.

**Figure 1:**
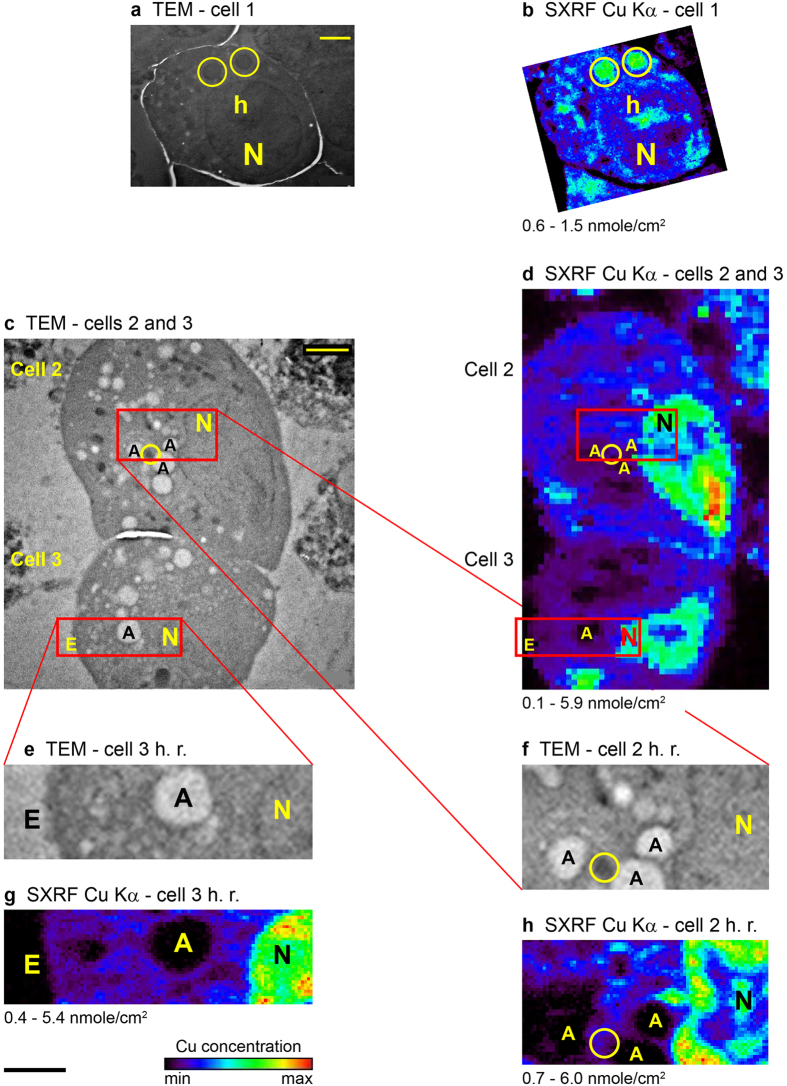
Complementary TEM and SXRF micro/nanoprobe Cu Kα distribution maps of MIN6 cells 1–3. The combined information from the two imaging techniques gives a detailed ultrastructural and chemical picture of the subcellular environment at the single organelle level. (**a**) TEM map of cell 1. This cell was grown in a medium with no Cd. It serves as a reference for the sample preparation and imaging procedure we used. Scale bar = 2 μm. (**b**) The corresponding SXRF microprobe map to a. Scan step size = 0.1 μm, dwell time = 1 s. Same scale as a. The two Au nanoparticles that are seen in the map, as red pixels in the upper and lower left parts, are off the reported concentration range. (**c**) TEM image of cells 2 and 3. These cells were grown in a medium supplemented with 1 μmole/l CdCl_2_. Red rectangles outline the areas imaged with the SXRF nanoprobe at high-resolution. Scale bar = 2 μm. (**d**) The SXRF microprobe map corresponding to c. Scan step size = 0.25 μm, dwell time = 1 s. Same scale as c. (**e–f**) Enlargements of the high-resolution (h. r.) scan areas in the TEM image of cells 3 and 2 (**c**), respectively. (**g–h**) The SXRF nanoprobe maps corresponding to e–f. Scan step size = 0.05 μm, dwell time = 5 s. One μm scale bar (below **g**) applies to (**e–h**). Elemental spatial density scale bar (below g) applies to (**b**,**d**,**g**–**h**) and the measured range, in units of nmole/cm^2^, is indicated below each map. Contrast of the SXRF images (**b**,**d**,**g**–**h**) has been enhanced. A – autophagosome-like feature, E – edge of the cell, h – heterochromatin, N – nucleus, circles outline mitochondria. Additional concentration information, including uncertainties, appears in Supplementary Table S2.

**Figure 2:**
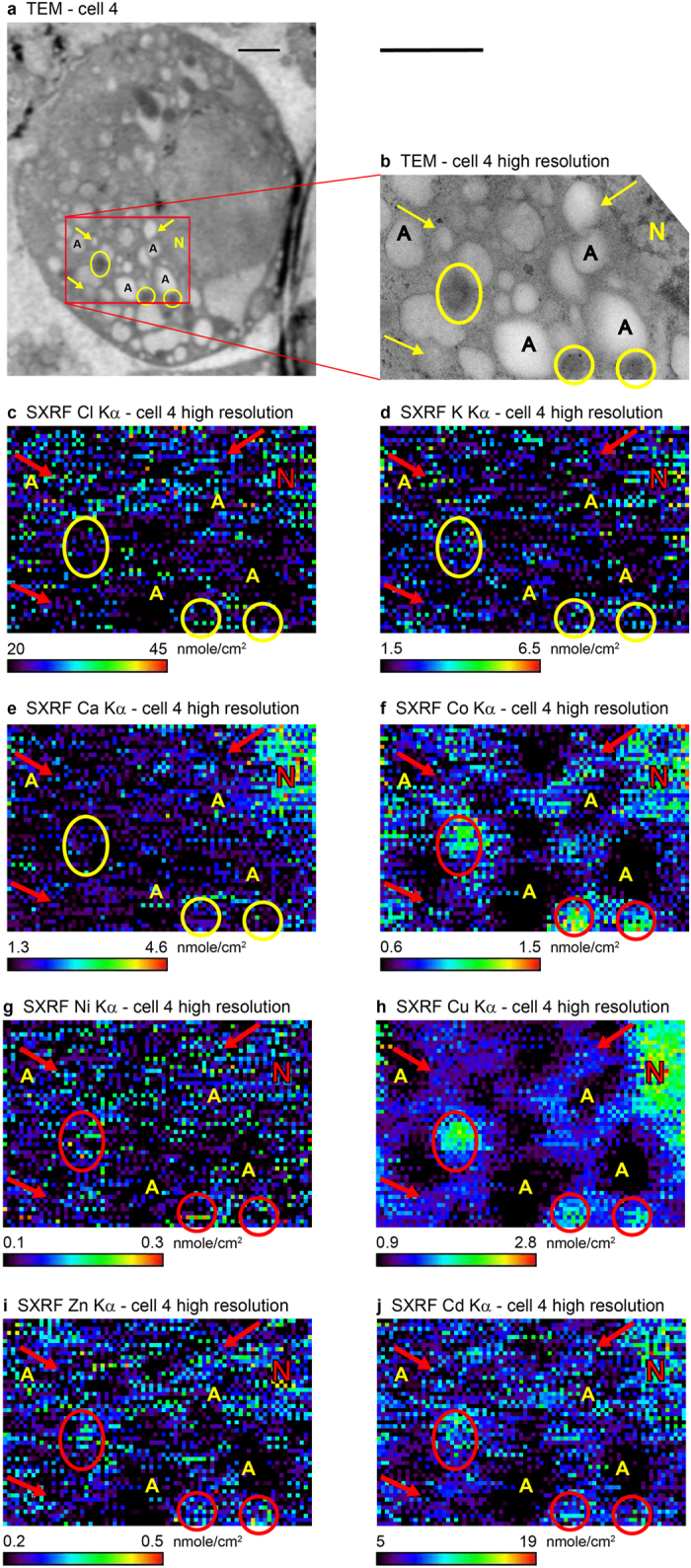
Complementary TEM and SXRF nanoprobe images of MIN 6 cell 4. The cell was grown in a medium supplemented with 1 μmole/l CdCl_2_. (Compare with untreated cell 5, Supplementary Fig. S1, Table S3). In this case, the 350 nm thick cell section was mounted on a non-coated Au TEM finder grid and was sandwiched between two Formvar layers (each ~100 nm thick). (**a**) TEM image of the cell. The red rectangle outlines the area imaged with the SXRF nanoprobe. Scale bar = 1 μm. (**b**) Enlargement of the high-resolution scan area in the TEM image a. (**c–j**) SXRF nanoprobe elemental distribution maps corresponding to b of, respectively, Cl, K, Ca, Co, Ni, Cu, Zn, Cd. All elemental spectra were collected simultaneously. With the exception of Cd, all elements were mapped by their Kα lines. Cadmium was mapped by its Lα lines. Scan step size = 0.04 μm, dwell time = 8 s. One μm scale bar (above **b**) applies to (**b–j**). Elemental spatial density scale bar with the measured range, in units of nmole/cm^2^, appears below each elemental map. Contrast of the SXRF images (**c–j**) has been enhanced. The Cl concentration, Kα_1,2_ = 2.622, 2.621 keV, respectively, in c may be overestimated due to the presence of an unidentified strong line at ~2.400 keV. Due to lack of suitable Cd standard, there could be a systematic uncertainty of up to a factor of 2 in the inferred Cd concentrations (j, see Methods). A – autophagosome-like feature, N – nucleus, arrows point to vesicles, potentially insulin producing, circles/ellipses outline mitochondria. Additional concentration information, including uncertainties, appears in Supplementary Table S2.

The cryo-fixed and plasticized cell samples were ultramicrotomed to a thickness of 350 nm. This thickness was chosen to optimize the requirements of SXRF and TEM, as well as the need to image single organelles. Thicker samples are preferable for SXRF imaging since the elemental X-ray signal intensity increases with sample thickness. At the same time, 350 nm is the maximum thickness that can be imaged by TEM and maintain spatial resolution. In addition, in order to chemically analyze single organelles by SXRF, the primary X-ray beam needs to traverse only one organelle at each point on the sample. Considering the tens to hundreds of nm size range and spatial distribution of organelles in the cells (Figs 1 and 2, S1), it is reasonable that this requirement was met in most cases.

### Transmission electron microscopy (TEM) and synchrotron X-ray fluorescence (SXRF) imaging

The cell sections were placed on Au TEM finder grids coated with carbon and Formvar. Whole sections were imaged by TEM and suitable cells for SXRF analysis were selected. The criteria for suitable cells were good preservation of cellular ultrastructure, no ice damage, and presence of a variety organelle types and other subcellular features (Figs. 1, 2, S1). The chemical compositions of the selected cells were then imaged with a 10 keV primary X-ray beam using the microprobe^32^ at beamline 2-ID-D of the Advanced Photon Source (APS) at Argonne National Laboratory (ANL). The microprobe has a 150 × 150 nm^2^ beam spot size on the sample and a flux of 4 × 10^9^ photons/s/0.01% BW (at 10 keV). These properties enable one to image whole cell sections in relatively short time. The whole cell scans give the chemical compositions of the sections and reveal significant elemental distributions within the sections (Figs. 1b, d). Areas within the cell sections that met the criteria above and gave good SXRF spectra were then selected for high-resolution nanoprobe SXRF imaging (the whole cell scans also helped with navigation on the cell sections during the high-resolution scans).

High spatial resolution and high sensitivity imaging of the preselected subcellular areas were performed with the ANL’s Center for Nanoscale Materials (CNM) nanoprobe^33^ (beamline 26-ID-C at the APS). The nanoprobe offers the highest available SXRF spatial resolution, with a 40 × 40 nm^2^ beam spot size and a flux of 1.8 × 10^9^ photons/s/0.01% BW (at 9.75 keV) (work is currently being done to improve the spatial resolution even further^34^,^35^). Figure 1 shows complementary TEM, microprobe and nanoprobe SXRF Cu Kα images of the same cells. The Cu distribution in the micro- and nano-SXRF maps, with spatial resolutions of <150 nm and 50 nm, respectively, can be clearly correlated with the ultrastructure seen in the TEM images. For example, high Cu content spots in the micro-SXRF image of cell 1 (Fig. 1b) correlate with mitochondria and heterochromatin (that is known to have Cu-containing proteins) in the cell’s TEM image (Fig. 1a). The high-resolution scans of areas in cells 2 and 3 (Figs. 1g, h) show very detailed maps that correlate with fine ultrastructure in Figs. 1e, f, including the nuclei, mitochondria, autophagosome-like features, and the edge of one of the cells. The nano-SXRF maps even reveal additional ultrastructure not seen in the TEM maps, as in the nuclei of the two cells. In addition, the Cu concentrations in cells 2 and 3 measured with the nanoprobe (Figs. 1g, h) agree with those measured with the microprobe (Fig. 1d). This demonstrates the robustness of the SXRF results obtained in two beamlines.

The TEM and high-resolution SXRF images of cell 4 are shown in Fig. 2. In this case, the sensitivity of the high-resolution SXRF imaging, with spatial resolution of 0.04 μm, is demonstrated by the distribution maps of seven other elements in addition to Cu: Cl, K, Ca, Co, Ni, Zn and Cd (elemental concentrations were below the TEM chemical imaging detection limit). All elemental spectra were collected simultaneously. The detection of Cd was especially challenging, due to its suggested low concentration in single organelles under these exposure conditions^36^ and since unlike the other elements above, which were detected by their K lines, it was detected by the much weaker L lines. However, due to lack of suitable Cd standard, there could be a systematic uncertainty of up to a factor of 2 in the inferred Cd concentrations (see Methods). In addition, the measured Cl concentration in this cell, using Kα_1,2_ = 2.622, 2.621 keV, respectively, may be overestimated due to the presence of an unidentified strong line at ~2.400 keV. (This unidentified line masked the S lines, with Kα_1,2_ = 2.308, 2.307 keV, respectively, and prevented unequivocal S detection).

As stated above, the cryo-fixation/freeze substitution method used to prepare the samples is the best for preserving the cell’s ultrastructure and elemental distributions. Hence, it is expected that the elemental distribution maps (Figs. 1, 2, S1) represent the natural distributions in living MIN6 cells. To evaluate whether our results are biologically realistic, we used the elemental spatial densities per pixel as a rough comparison with other SXRF studies^25^,^27^,^37^,^38^,^39^,^40^,^41^. These studies were conducted at different synchrotrons, used a variety of cell types, sample preparations and experimental conditions (all imaged cells at lower spatial resolutions than presented here). The comparison shows that our measured ranges for all elements are comparable to the ranges reported in the studies above. The only exception may be Co, for which our values are higher. This may indicate that our cells were contaminated with Co. However, since only a few studies measured Co by SXRF in single cells, it is unclear at this time whether our cells were contaminated or not. We tested as well whether treating cells with Cd (1 μmole/l CdCl_2_) affects other elements’ distributions and spatial densities. For this purpose, the data of cell 4 (Fig. 2, Table S2), which was treated with Cd, were compared with the data of cell 5 (Fig. S1, Table S3), which was not treated. Using again the range of elemental spatial densities per pixel, the comparison shows that the elemental concentration ranges in both cells are comparable, with the exception of Cd, as expected. This indicates that the elemental distributions and concentrations at the cellular level were not affected by exposure to Cd at the level of 1 μmole/l CdCl_2_. In addition, the cellular ultrastructure of the cells we analyzed appears free of processing artifacts in the TEM images. However, despite the agreement with other studies, considering the natural variability of cell elemental compositions, it cannot be ruled out that the original elemental compositions and distributions of the cells we analyzed were altered to some extent.

## Discussion

The comparison between the TEM images and high-resolution SXRF elemental maps in Fig. 2 reveals some interesting initial observations regarding the natural elemental distributions between the different organelles. Divalent metals that are known to bind to proteins, mainly Co, Cu, Zn, Cd, and potentially Ni, have similar distributions. They are concentrated in areas rich in proteins, such as the nucleus and mitochondria, while having lower concentrations in the autophagosome/lysosome-like structures. The likely reason for the latter is that the affinity of divalent metals for proteins is significantly lower in lower pH environments, such as that in the autophagosome/lysosome, which leads to their dissociation from the proteins. Chlorine and K, on the other hand, have lower affinities for cellular proteins. The Cl^−^ and K^+^ ions are mainly present in cells in free solution. This is reflected in their more even distribution between the organelles. The chemical behavior of Ca is in between those of the two groups of elements above. The concentrations of the less tightly bound elements, Cl and K, are more susceptible to alteration during sample preparation, which could lead to differences between measured and natural concentrations and distributions.

These observations agree qualitatively with the results of Matsuyama *et al*.^25^. These authors combined fluorescence optical microscopy and nano-SXRF, with spatial resolution of 70 nm, to map elemental distributions in whole, chemically fixed, NIH/3T3 cells. Similar to the results presented here, Matsuyama *et al*. found as well that Cl, Ca, Cu and Zn (as well as P, S and Fe which are not reported here) were concentrated in the nucleus, and that Zn was concentrated as well in mitochondria.

The method presented here enables simultaneous mapping of subcellular distributions of multiple elements non-destructively at high spatial resolution and high sensitivity, and without the need for fluorescent indicators. It offers higher spatial resolution than fluorescence optical microscopy and since it does not use fluorescent indicators, avoids biases in the way they bind to the target elements and minimizes the potential for altering the natural elemental distributions. This was enabled by improved sample preparation technique, imaging thin (350 nm) cell sections, which ensure that in most cases the primary X-ray beam transverses only one organelle at each spot on the sample, combining TEM, micro- and nano-SXRF imaging, and the high spatial resolution (≤40 nm) and elemental detection sensitivity of the nanoprobe. We demonstrated the capabilities of the method by mapping unequivocally for the first time, to the best of our knowledge, natural elemental distributions, including those of some trace elements, between single organelles and other subcellular compartments.

An example of where the method presented here could provide insights not available from other analytical techniques is the role of Zn in insulin producing pancreatic beta cells. Zinc plays a prominent role in the proper function of these cells through several unique mechanisms^4^,^42^,^43^,^44^,^45^,^46^. It is co-secreted with insulin and therefore a high cellular turnover of Zn has to be maintained. Zinc homeostasis is achieved through the interaction between Zn and several specialized transporter (ZnT and ZIP family) and buffering proteins, mainly metallothionein, as well as regulated transcription of the proteins involved in Zn trafficking and buffering^5^,^47^,^48^,^49^,^50^,^51^,^52^,^53^,^54^,^55^,^56^,^57^,^58^,^59^. The relevance of Zn homeostasis to the development of diabetes mellitus has been highlighted by numerous studies. They linked a common, non-silent genetic variation in the gene encoding the beta cell specific Zn transporter protein ZnT8 to beta cell dysfunction and type 2 diabetes mellitus^60^,^61^,^62^,^63^,^64^,^65^,^66^. The exact mechanism by which alterations in this Zn transporter protein leads to beta cell dysfunction has remained elusive so far, despite intense efforts to decipher its action^4^,^45^,^67^,^68^. Specifically, the mechanism of Zn transfer from its main buffering protein, metallothionein, to ZnT8 and other Zn acceptor proteins is being studied extensively. The mechanism by which the presence of Zn in most mammalian insulin producing vesicles facilitates the proper maturation of insulin precursors to biologically active insulin is an additional area of active investigation^42^,^43^. A better understanding of the distributions of protein-bound Zn and other, potentially interfering, divalent metals in various subcellular compartments of beta cells, which could be studied using the method presented here, is essential for understanding the role of Zn homeostasis in the development of diabetes mellitus.

Two recent papers^69^,^70^ showed that analyzing single cells reveals important metabolomic information that is not available from cell population level studies. Similarly, the method presented here, which could likely be applied to every cell type, opens up new possibilities for studies at the single organelle level, as in the Zn homeostasis example above. Such studies will provide new insights which are not available from organelle population level studies and help answer many important biological questions related to the biochemistry of living cells.

## Methods

### Cells and culture

The MIN6 cells were a gift from Dr. Junichi Miyazaki (Osaka University Medical School, Osaka, Japan). They were grown and maintained as described previously^28^,^36^. Briefly, cells were plated on 6 well cell culture dishes (Corning) at a density of 0.8 × 10^6^ cells per well. The cell culture medium was composed of DMEM (Invitrogen) supplemented with 15% heat inactivated FBS (Hyclone), 100 U/ml penicillin, 100 μg/ml streptomycin (Invitrogen), 2 mmole/l glutamine (Invitrogen) and 50 μM beta mercaptoethanol (Invitrogen). Cells between passages 28 and 35 were used for the experiments. Cells were grown at 5% CO_2_ and 37 °C. Culture medium was exchanged at 48 hours following cell seeding. At that time, 1.0 μmole/l CdCl_2_ or vehicle (PBS) were added.

### Sample preparation and transmission electron microscopy (TEM) imaging

Cells were harvested 72 hours after being placed into planchettes (Ted Pella), cryo-fixed using a high pressure freezing machine (HPM 010; RMC), and placed immediately into cryo-tubes containing a frozen cocktail of 0.25% glutaraldehyde and 0.1% tannic acid in anhydrous acetone. All frozen samples were placed into an automatic freeze substitution device (ASF2, Leica). The samples were freeze substituted at −80 °C for 48 hrs, then the temperature was raised to −50 °C. The samples were washed 3 times with acetone, infiltrated with successive increasing concentrations (25%, 50%, 75%, each 8 hrs long; 100% overnight) of Lowicryl HM20 resin, followed by three incubations, 1 hr each, with 100% resin. The samples were UV polymerized at −50 °C for 20 hrs. 350 nm sections were cut with an ultramicrotome (UC6, Leica) and placed on a gold, Formvar-carbon coated, London-finder grid (EMS). Images were collected on an electron microscope operated at 300 kV (Tecnai TF30; FEI) with a 4K ultrascan digital camera (Gatan). Cells were selected for SXRF imaging based on two criteria: (a) showing no ice damage, and (b) showing variety of well-preserved organelles.

### Synchrotron X-ray fluorescence (SXRF) imaging

The SXRF chemical imaging was performed at the Advanced Photon Source (APS) at Argonne National Laboratory (ANL). Whole, TEM pre-selected, cell sections were imaged with the microprobe at beamline 2-ID-D. Based on the TEM and microprobe SXRF images, areas within cell sections, with a variety of well-preserved organelles and good SXRF spectra, were selected for high-resolution SXRF imaging with the Center for Nanoscale Materials (CNM) nanoprobe (beamline 26-ID-C). The SXRF spectra from both beamlines were quantified and visualized with the MAPS software package^71^.

The primary X-ray beam energy in both beamlines was 10 keV. All elements analyzed, with the exception of Cd, were detected by their K lines. The standards used for these elements were Standard Reference Materials (SRM) 1832 and 1833 by the National Institute of Standards and Technology (NIST). Cadmium was detected by its L lines. We used standards with varying Cd/Zn ratios to improve Cd quantification by correcting (1) relative Cd L line intensities, and (2) normalization of Cd L line intensities to Zn K line intensities. The standards were prepared by depositing drops of liquids with varying Cd/Zn ratios on Formvar-coated TEM slot grids (EMS) (one Cd/Zn ratio per grid). When the liquids evaporated, they left residues on the Formvar with different Cd/Zn ratios. The standards worked well for correcting relative Cd L line intensities. However, The Cd/Zn ratios varied spatially on the grids and did not match the nominal ratios in the liquids, so they could not be used to normalize the Cd/Zn concentrations. As a consequence, there could be a systematic uncertainty of up to a factor of 2 in the inferred Cd concentrations in the cells. We are planning to reduce the uncertainty in quantifying the Cd concentration in future measurements by using improved standards.

## Additional Information

**How to cite this article**: Kashiv, Y. *et al*. Imaging trace element distributions in single organelles and subcellular features. *Sci. Rep*. **6**, 21437; doi: 10.1038/srep21437 (2016).

## Supplementary Material

Supplementary Information
