# Supplementary material for: Imaging trace element distributions in single organelles and subcellular features
Source: Sci Rep. 2016 Feb 25;6:21437. doi: 10.1038/srep21437 (PMC4766485; doi:10.1038/srep21437)
Supplement: Supplementary Information [file srep21437-s1.pdf]

# Imaging trace element distributions in single organelles and subcellular features

Yoav Kashiv[1], Jotham R. Austin II[2], Barry Lai[3], Volker Rose[3,4], Stefan Vogt[3], Malek El-Muayed[5]

[1]*Department of Physics, University of Notre Dame, Notre Dame, IN 46556-5670*

[2]*Advanced Electron Microscopy Facility, Department of Molecular Genetics & Cell Biology, The University of Chicago, Chicago, IL 60637*

[3]*X-Ray Science Division, Argonne National Laboratory, Argonne, IL 60439*

[4]*Center for Nanoscale Materials, Argonne National Laboratory, Argonne, IL 60439*

[5]*Division of Endocrinology, Metabolism and Molecular Medicine, Northwestern University Feinberg School of Medicine, Chicago, IL 60611*

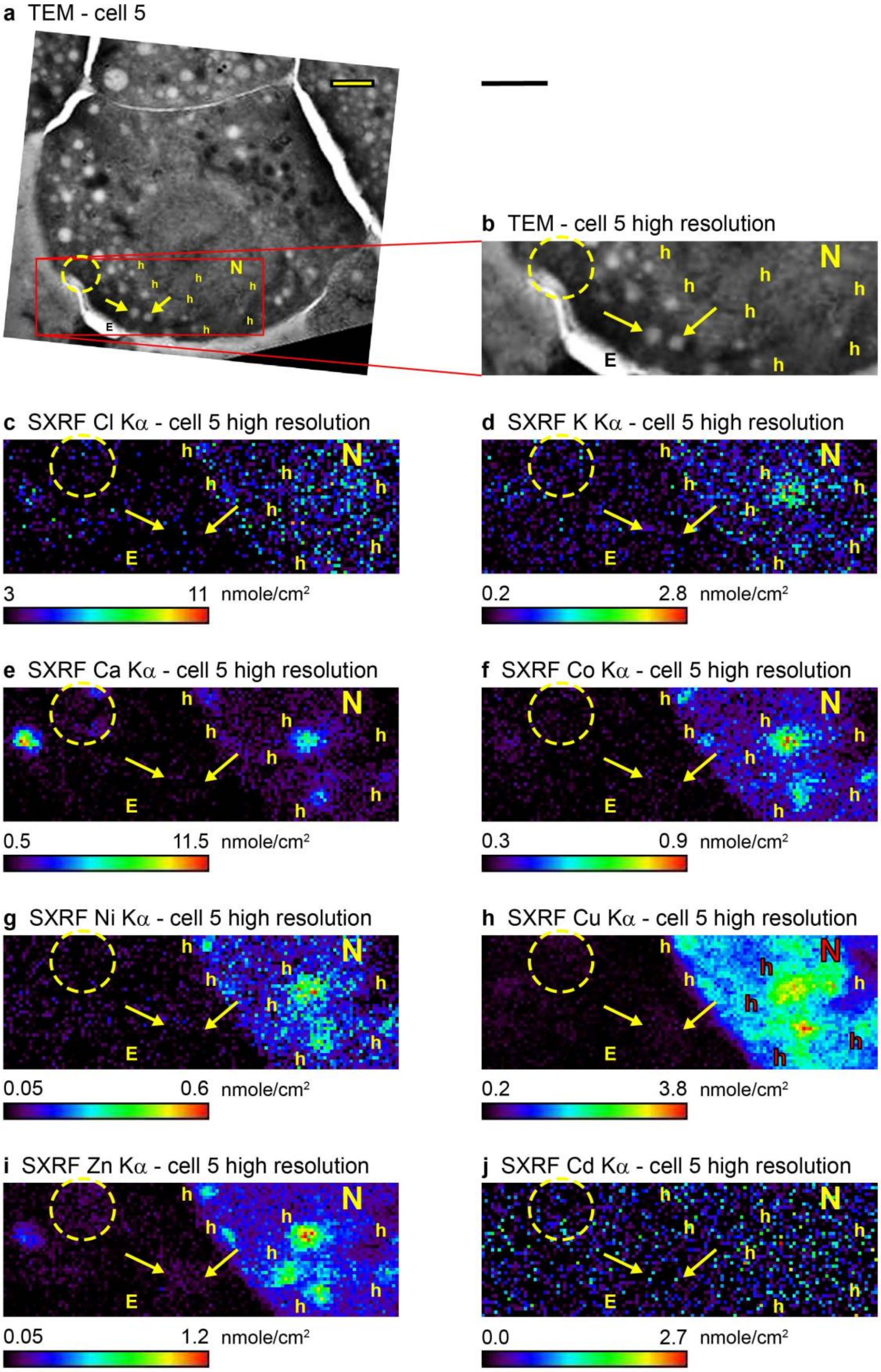

a TEM - cell 5
b TEM - cell 5 high resolution
c SXRF Cl Kα - cell 5 high resolution
d SXRF K Kα - cell 5 high resolution
e SXRF Ca Kα - cell 5 high resolution
f SXRF Co Kα - cell 5 high resolution
g SXRF Ni Kα - cell 5 high resolution
h SXRF Cu Kα - cell 5 high resolution
i SXRF Zn Kα - cell 5 high resolution
j SXRF Cd Kα - cell 5 high resolution
N
h
E
3 11 nmole/cm2
0.2 2.8 nmole/cm2
0.5 11.5 nmole/cm2
0.3 0.9 nmole/cm2
0.05 0.6 nmole/cm2
0.2 3.8 nmole/cm2
0.05 1.2 nmole/cm2
0.0 2.7 nmole/cm2

**Figure S1. Complementary TEM and SXRF nanoprobe images of MIN 6 cell 5.** The cell was grown in a medium not supplemented with Cd and it serves as a non-treated cell reference to treated-cell 4 (Fig. 2). As with cells 1-3 (Fig. 1), the 350 nm thick cell 5 section was placed on a Au TEM finder grids coated with carbon and Formvar. (**a**) TEM image of the cell. The red rectangle outlines the area imaged with the SXRF nanoprobe. Scale bar = 1 μm. (**b**) Enlargement of the high-resolution scan area in the TEM image **a**. (**c - j**) SXRF nanoprobe elemental distribution maps corresponding to **b** of, respectively, Cl, K, Ca, Co, Ni, Cu, Zn, Cd. All elemental spectra were collected simultaneously. With the exception of Cd, all elements were mapped by their Kα lines. Cadmium was mapped by its Lα lines. Scan step size = 0.05 μm, dwell time = 4 s. One μm scale bar (above **b**) applies to **b** – **j**. Spatial density scale bar with the measured range, in units of $nmole/cm^2$, appears below each elemental map. Contrast of the SXRF images (**c-j**) has been enhanced. Due to lack of a suitable Cd standard, there could be a systematic uncertainty of up to a factor of 2 in the inferred Cd concentrations (**j**, see Methods). E – edge of the cell, h – heterochromatin, N – nucleus, arrows point to vesicles, dashed circle outlines a group of vesicles; all vesicles are potentially insulin producing. Additional concentration information, including uncertainties, appears in Table S3. Three important observations emerge from the comparison between the elemental distribution maps in Figs. 2 and S1, and the additional information in Tables S2 (Fig. 2) and S3 (Fig. S1):

1. With the exception of Cd, the spatial density ranges of each element are comparable in both figs. This indicates that supplementing cells with 1 μmole/l $CdCl_2$ did not affect other elements' concentration ranges at the cellular level.

2. As expected, the maximum concentration of Cd in the non-supplemented cell 5 (Fig. S1j) is significantly lower than in the Cd-supplemented cell 4 (Fig. 2j). In addition, since Cd is a biologically non-essential element that is naturally present in cells at very low concentrations, cell 5's ultrastructure (Fig. S1b) cannot be identified in the Cd distribution map (Fig. S1j).

3. The small difference in each element's distribution map minimum values between cell 4 (Fig. 2) and cell 5 (Fig. S1) is mainly due to the different contrasts used in in each fig. The data in Tables S2 and S3 show that the absolute minimum vales for each element are comparable in both figs.

**Table S1. Elemental analysis of chemicals used in sample preparation[a] and comparison with maximum values measured in cell 4 (Fig. 2, Table S2).** Concentrations in the chemicals were measured by inductively coupled plasma mass spectrometry (ICP-MS) and are reported in units of nmole/l ± 1σ. The negligible elemental concentrations in the chemicals, compared with the max concentrations per pixel measured in the cell, makes cells contamination by the chemicals very unlikely.

| | Mg | | | Mn | | | Fe | | | Co | | |
|---|---|---|---|---|---|---|---|---|---|---|---|---|
| Water | 531 | ± | 214 | 5.28 | ± | 0.07 | 138 | ± | 47 | 0.00 | + | 0.04 |
| Acetone | 73 | ± | 3 | 11.721 | ± | 0.023 | 974 | ± | 22 | 0.00 | + | 0.07 |
| Gluteraldehyde | 1397 | ± | 15 | 19.27 | ± | 0.10 | 495 | ± | 11 | 0.00 | + | 0.08 |
| Tanic acid | 4553.1 | ± | 2.8 | 39.1 | ± | 0.3 | 962 | ± | 15 | 0.00 | + | 0.13 |
| HM20 | 484 | ± | 15 | 18.4 | ± | 0.6 | 738 | ± | 19 | 0.00 | + | 0.13 |
| Ethylene glycol | 1081 | ± | 50 | 19.3 | ± | 0.3 | 379 | ± | 32 | 0.00 | + | 0.14 |
| Cell max[b] | | - | | | - | | | - | | $58\times10^6$ | ± | $6\times10^6$ |

**Table S1.  Continued.**

| | Ni | | | Cu | | | Zn | | |
|---|---|---|---|---|---|---|---|---|---|
| Water | 6.7 | ± | 0.7 | 14.4 | ± | 2.7 | 275 | ± | 7 |
| Acetone | 10.0 | ± | 1.1 | 14.0 | ± | 1.3 | 340.7 | ± | 2.3 |
| Gluteraldehyde | 24.3 | ± | 0.8 | 30.1 | ± | 1.0 | 1748 | ± | 11 |
| Tanic acid | 80.0 | ± | 0.6 | 205 | ± | 5 | 2700 | ± | 4 |
| HM20 | 98.6 | ± | 1.3 | 68.6 | ± | 1.3 | 740 | ± | 4 |
| Ethylene glycol | 24.0 | ± | 1.1 | 14.2 | ± | 1.3 | 615.7 | ± | 2.4 |
| Cell max[b] | $10.0\times10^{6}$ | ± | $2.4\times10^{6}$ | $81\times10^{6}$ | ± | $6\times10^{6}$ | $15.6\times10^{6}$ | ± | $2.5\times10^{6}$ |

**Table S1.  Continued.**

| | As | | | Se | | | Y | | |
|---|---|---|---|---|---|---|---|---|---|
| Water | 0 | + | 24 | 9 | +<br>- | 13<br>9 | 267.0 | ± | 1.8 |
| Acetone | 24 | ± | 7 | 0.2 | +<br>- | 1.9<br>0.2 | 194.2 | ± | 0.7 |
| Gluteraldehyde | 19 | +<br>- | 23<br>19 | 0.0 | + | 2.9 | 194.4 | ± | 2.9 |
| Tanic acid | 7 | +<br>- | 17<br>7 | 0 | + | 13 | 216.1 | ± | 0.7 |
| HM20 | 65 | ± | 19 | 15 | ± | 14 | 203.13 | ± | 0.28 |
| Ethylene glycol | 1384 | ± | 41 | 284 | ± | 18 | 196.4 | ± | 2.0 |
| Cell max[b] | | - | | | - | | | - | |

**Table S1. Continued.**

| | Cd[c] | | | In | | | Ho | | | Pb | | |
|---|---|---|---|---|---|---|---|---|---|---|---|---|
| Water | 0.50 | ± | 0.14 | 205.4 | ± | 1.2 | 146.1 | ± | 0.3 | 2.58 | ± | 0.05 |
| Acetone | 0.155 | ± | 0.017 | 149.0 | ± | 0.9 | 107.8 | ± | 0.4 | 0.40 | ± | 0.08 |
| Gluteraldehyde | 0.60 | ± | 0.08 | 150.3 | ± | 1.5 | 108.3 | ± | 0.8 | 1.960 | ± | 0.003 |
| Tanic acid | 2.49 | ± | 0.16 | 166.5 | ± | 1.1 | 120.5 | ± | 1.2 | 100.0 | ± | 0.6 |
| HM20 | 0.40 | ± | 0.07 | 156.0 | ± | 0.3 | 111.9 | ± | 0.4 | 48.0 | ± | 0.3 |
| Ethylene glycol | 0.86 | ± | 0.05 | 152.9 | ± | 1.4 | 109.2 | ± | 0.9 | 0.73 | ± | 0.08 |
| Cell max[b] | $569 \times 10^6$ | ± | $75 \times 10^6$ | - | | | - | | | - | | |

[a] Elemental concentrations measured by ICP-MS in the chemicals were calculated from the following isotopic measurements:

Mg: weighted average of $^{24}$Mg and $^{26}$Mg.
Mn: $^{55}$Mn.
Fe: $^{57}$Fe.
Co: $^{59}$Co.
Ni: weighted average of $^{58}$Ni and $^{60}$Ni.
Cu: $^{65}$Cu.
Zn: weighted average of $^{64}$Zn and $^{66}$Zn.
As: $^{75}$As.
Se: $^{79}$Se.
Y: $^{89}$Y.
Cd: weighted average of $^{111}$Cd and $^{112}$Cd.
In: $^{115}$In.
Ho: $^{165}$Ho.
Pb: $^{208}$Pb.

[b] Cell max is the value of the pixel with the highest elemental concentration. Maximum concentrations (volume densities) are calculated from the maximum elemental spatial densities in Supplementary Table S2 by $C \approx S/T*f$, where C is the concentration (nmole/l), S is the spatial density in Table S2 (nmole/cm$^2$), T is the sample thickness (350 nm = $3.5\times10^{-5}$ cm), f is the nmole/cm$^3$ to nmole/l conversation factor (=$10^3$). The calculated concentrations are average values, assuming that the elements are evenly distributed throughout the sample thickness.

[c] Due to lack of a suitable Cd standard, there could be a systematic uncertainty of up to a factor of 2 in the inferred Cd concentrations (**j**, see Methods).

**Table S2. Additional elemental concentration information for Figs. 1 and 2.** Concentrations are reported as spatial densities in units of nmole/cm$^2$ ± 1σ.

| Figure | Element | Black pixel range | | Red pixel range | |
|---|---|---|---|---|---|
| | | min | max | min | max |
| 1 | | | | | |
| b | Cu | 0.079 ± 0.015 | 0.64 ± 0.04 | | 1.50 ± 0.07 |
| d | Cu | 0.070 ± 0.012 | | | 5.93 ± 0.11 |
| g | Cu | 0.13 ± 0.04 | 0.38 ± 0.06 | | 5.35 ± 0.23 |
| h | Cu | 0.29 ± 0.05 | 0.68 ± 0.08 | | 6.00 ± 0.24 |
| 2 | | | | | |
| c | Cl | 0.00 + 0.26 | 19 ± 3 | | 45 ± 5 |
| d | K | 0.00 + 0.07 | 1.5 ± 0.6 | | 6.5 ± 1.2 |
| e | Ca | 0.00 + 0.07 | 1.3 ± 0.5 | | 4.6 ± 0.9 |
| f | Co | 0.09 ± 0.05 | 0.62 ± 0.12 | 1.47 ± 0.19 | 2.02 ± 0.22 |
| g | Ni | 0.000 + 0.017 | 0.09 ± 0.04 | 0.31 ± 0.08 | 0.35 ± 0.08 |
| h | Cu | 0.19 ± 0.06 | 0.87 ± 0.12 | | 2.82 ± 0.21 |
| i | Zn | 0.036 ± 0.022 | 0.22 ± 0.05 | 0.51 ± 0.08 | 0.54 ± 0.09 |
| j | Cd | 0.00 + 0.21 | 5.5 ± 1.4 | 19.4 ± 2.6 | 19.9 ± 2.6 |

Due to enhanced contrast in the elemental maps in Figs. 1 and 2, minimum values (black pixels) represent a range of spatial densities in all figs., except in Fig. 1d. Similarly, in Figs. 2f, g, i, j, maximum values (red pixels) represent a range of spatial densities. In cases where only one min/max concentration value is reported (min for black pixels or max for red pixels), the black/red pixels correspond only to this value and not to a range of values.

Uncertainties are calculated from counting statistics. They do not include uncertainties due to background subtraction (which makes a small contribution to the total uncertainty of most elements in the table) and

potential systematic uncertainties. Calculating uncertainty for spatial density = 0 is difficult. The uncertainties in these cases are calculated from the lowest measured non-zero spatial densities in the maps. This means that the reported values in these cases are lower limits on the uncertainties.

We did not have a suitable standard for normalizing Cd concentrations, measured in this study by the Cd L lines, to concentrations measured by K lines (all other elements reported here). As a consequence, there could be a systematic uncertainty of up to a factor of 2 in the calculated Cd concentrations (see Methods).

**Table S3. Additional elemental concentration information for Fig. S1.** Concentrations are reported as spatial densities in units of nmole/cm$^2$ ± 1$\sigma$.

| Figure | Element | Black pixel range | | Red pixel range | |
|---|---|---|---|---|---|
| | | **min** | **max** | **min** | **max** |
| c | Cl | 0.00 + 0.05 | 3.0 ± 1.1 | | 11.0 ± 2.2 |
| d | K | 0.000 + 0.012 | 0.23 ± 0.20 | | 2.8 ± 0.7 |
| e | Ca | 0.000 + 0.010 | 0.46 ± 0.23 | | 11.6 ± 1.2 |
| f | Co | 0.000 + 0.007 | 0.35 ± 0.25 | 0.94 ± 0.13 | 1.19 ± 0.15 |
| g | Ni | 0.0000 + 0.0020 | 0.050 ± 0.027 | | 0.58 ± 0.09 |
| h | Cu | 0.031 ± 0.019 | 0.18 ± 0.05 | | 3.76 ± 0.21 |
| i | Zn | 0.000 + 0.007 | 0.047 ± 0.022 | | 1.16 ± 0.11 |
| j | Cd | 0.00 + 0.15 | | | 2.7 ± 0.8 |

Due to enhanced contrast in the elemental maps in Fig. S1, minimum values (black pixels) represent a range of concentrations in all figs., except in Fig. S1j. Similarly, in Fig. S1f, maximum values (red pixels) represent a range of concentrations. In cases where only one min/max concentration value is reported (min for black pixels or max for red pixels), the black/red pixels correspond only to this value and not to a range of values.

Uncertainties are calculated from counting statistics. They do not include uncertainties due to background subtraction (which makes a small contribution to the total uncertainty of most elements above) and potential systematic uncertainties. Calculating uncertainty for spatial density = 0 is difficult. The uncertainties in these cases are calculated from the lowest measured non-zero spatial densities in the maps. This means that the reported values in these cases are lower limits on the uncertainties.

We did not have a suitable standard for normalizing Cd concentrations, measured in this study by the Cd L lines, to concentrations measured by K lines (all other elements reported here). As a consequence, there could be a systematic uncertainty of up to a factor of 2 in the calculated Cd concentrations (see Methods).
